# Crystal structure of a homoleptic zinc(II) complex based on bis­(3,5-diiso­propyl­pyrazol-1-yl)acetate

**DOI:** 10.1107/S2056989018011246

**Published:** 2018-08-16

**Authors:** Josiah G. Elsberg, Nicholas G. Spiropulos, Adam C. Colson, Eric C. Brown

**Affiliations:** a Department of Chemistry and Biochemistry, Boise State University, 1910 University Drive, Boise, ID 83725, USA

**Keywords:** crystal structure, zinc, heteroscorpionate ligands

## Abstract

Deprotonation of the methyl­ene group in bis­(3,5-diiso­propyl­pyrazol-1-yl)methane with *n*BuLi and reaction with carbon dioxide yields lithium bis­(3,5-diiso­propyl­pyrazol-1-yl)acetate (**1**). Treatment of **1** with ZnCl_2_ results in the com­pound bis­[bis­(3,5-diiso­propyl­pyrazol-1-yl)acetato]­zinc(II), [Zn(C_20_H_31_N_4_O_2_)_2_] (**2**), whose structure has monoclinic (*P*2_1_/*c*) symmetry. The Zn^II^ ion resides on an inversion center and is coordinated by two bis­(3,5-diiso­propyl­pyrazol-1-yl)acetate (bdippza) ligands. Each ligand facially coordinates the zinc center *via* κ^3^ *N*,*N*′,*O* coordination modes to form a distorted octa­hedral complex with four pyrazole N atoms in the basal plane and two carboxyl­ate O atoms in the axial sites.

## Abstract 2

The compound [(bdippza)_2_Zn], where bdippza is bis­(3,5-diiso­propyl­pyrazol-1-yl)acetate, possesses inversion symmetry and the Zn^II^ atom is located on the special position of the *P*2_1_/*c* space group. The zinc atom is coordinated by two bdippza ligands resulting in a six-coordinate distorted octa­hedral geometry.

## Chemical context   

The closely related zinc-containing enzymes thermolysin (Holland *et al.*, 1995 ▸) and carb­oxy­peptidase A (Rees *et al.*, 1983 ▸) each contain an active site where a distorted tetra­hedral zinc ion is coordinated to two histidine residues, a glutamate residue, and a water mol­ecule. These enzymes catalyze the hydrolysis of peptide bonds containing hydro­phobic residues with thermolysin selective for the amide bonds located on the N-terminal side of the polypeptide (Heinrikson, 1977 ▸), while carb­oxy­peptidase A prefers the amide bonds on the C-terminal side (Lipscomb, 1970 ▸). However, questions remain concerning the mechanism of amide-bond hydrolysis by thermolysin and carb­oxy­peptidase A. As such, the synthesis and study of model complexes that mimic the active-site structure and reactivity of these biological compounds is necessary to their further understanding.

In an attempt to model the two histidine and glutamate binding motifs present in thermolysin and carb­oxy­peptidase A, the coordination chemistry of bis­(3,5-diiso­propyl­pyrazol-1-yl)acetate (bdippza) with zinc chloride was explored to determine if the steric demands of the anionic heteroscorpionate ligand were suitable to form a zinc complex of the form [(bdippza)ZnCl]. However, structural determination of the title compound identified the product not as the target compound but instead as the homoleptic zinc compound [(bdippza)_2_Zn] (**2**). Formation of **2** occurs regardless of the stoichiometric ratio and indicates that the steric environment of the bdippza ligand is too small to prevent complexation of two ligands per zinc ion. Spectroscopic characterization of **2** is consistent with the solid-state structure. For instance, identification of the acetate group is evident by a strong IR absorption at 1687 cm^−1^ and a ^13^C NMR signal at 165.8 ppm (the carbon peak of the carboxyl­ate was identified by an HMBC experiment that showed a two-bond correlation between the proton of the bridging C atom and the C atom of the carboxyl­ate). Furthermore, the positive-ion ESI–MS spectrum of **2** shows the presence of the [*M* + Na]^+^ ion, whose isotope pattern is in good agreement with the theoretical isotope pattern of the compound (see supporting information for ESI–MS spectra and 1D and 2D NMR spectra).
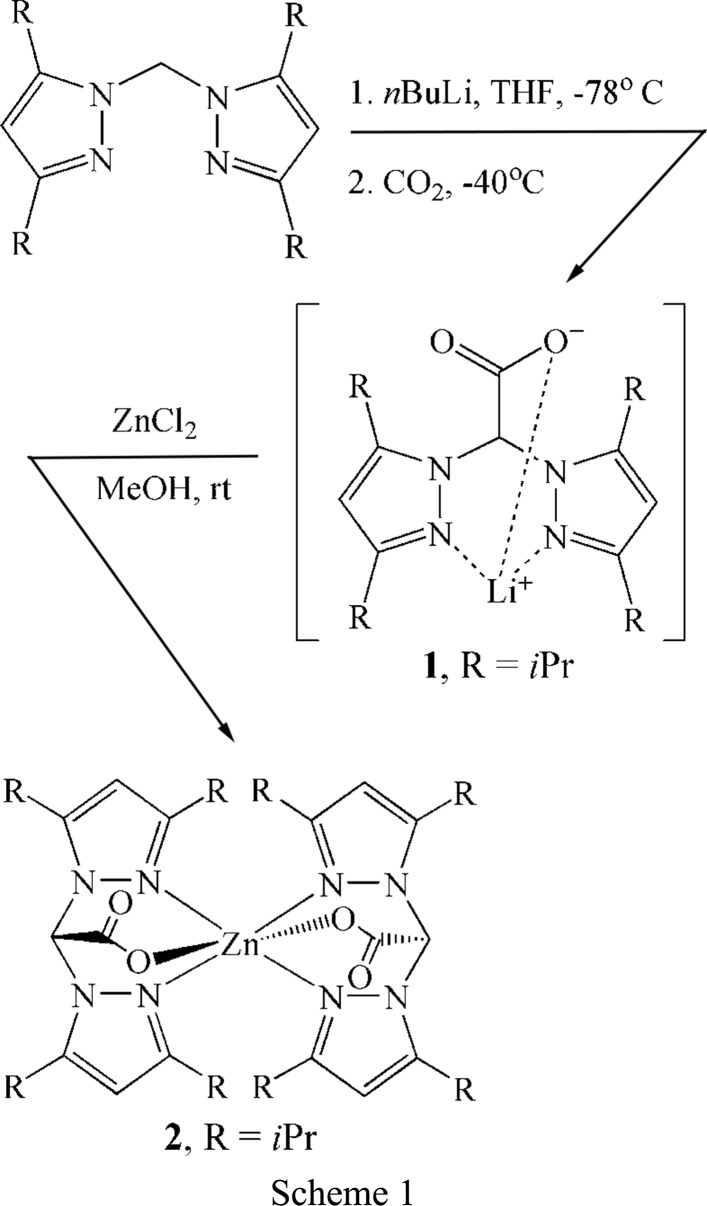


## Structural commentary   

**Figure 1:**
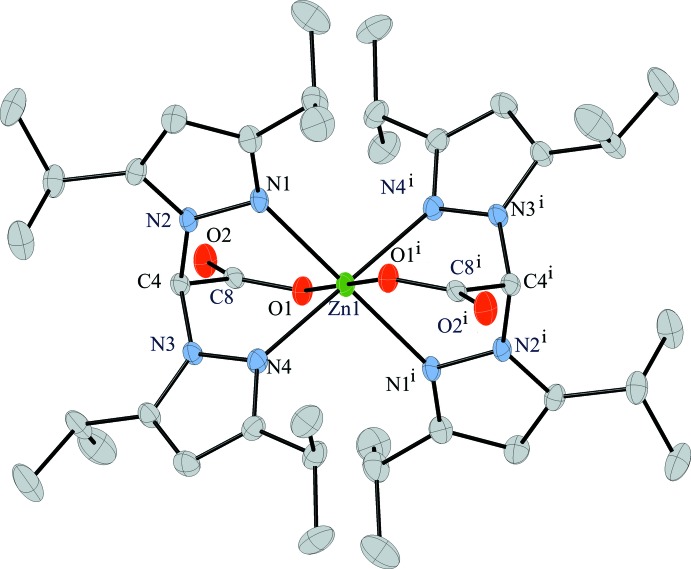
A view of the structure of the title compound, showing the atom-labeling scheme. Displacement ellipsoids are drawn at the 50% probability level. Symmetry code for generating equivalent atoms: (i) −*x*, −*y*, −*z* + 1.

The mol­ecular structure of the title complex is shown in Fig. 1 ▸. Selected bond lengths and angles are given in Table 1 ▸. The Zn^II^ ion resides on an inversion center and is coordinated by two bdippza ligands to form a six-coordinate complex. The two ligands facially bind the Zn^II^ ion in a tridentate fashion, with four N atoms making up the basal plane of the distorted octa­hedron and the two carboxyl­ate oxygens binding the Zn^II^ at the remaining apical positions in a *trans* manner (O—Zn—O angle of 180.0°). The Zn—N_pyrazole_ bond lengths range from 2.1674 (11) to 2.1942 (12) Å and the N—Zn—N angles of the basal plane range from 82.91 (4) to 97.09 (4)°. The apical O atoms are positioned approximately perpendicular to the basal plane, with angles that deviate slightly from 90° [O1—Zn1—N1 = 86.51 (4), O1—Zn1—N1^i^ = 93.49 (4), O1—Zn1—N4 = 86.40 (4) and O1—Zn1—N4^i^ = 93.60 (4)°]. The Zn—O bond length is 2.0471 (10) Å. The carbonyl oxygen of the carboxyl­ate donor is tilted away from the zinc carboxyl­ate plane, as indicated by the Zn1—O1—C8—C4 torsion angle of 20.61 (16)°. Complexation of the two bdippza ligands to the Zn^II^ ion results in the formation of six six-membered metallocycles [Zn1–O1–C8–C4–N2–N1 (A), Zn1–O1–C8–C4–N3–N4 (B), Zn1–N1–N2–C4–N3–N4 (C), Zn1–O1^i^–C8^i^–C4^i^–N2^i^–N1^i^ (D), Zn1–O1^i^–C8^i^–C4^i^–N3^i^–N4^i^ (E), and Zn1–N1^i^–N2^i^–C4^i^–N3^i^–N4^i^ (F)] that are all nonplanar. A ring-puckering analysis [puckering parameters are: *Q* = 0.9102 (12), θ = 85.76 (8)°, ψ = 346.77 (8)° for A, *Q* = 0.8809 (11), θ = 96.27 (8)°, ψ = 190.62 (8)° for B, *Q* = 0.9932 (11), θ = 80.32 (7)°, ψ = 350.91 (7)° for C, *Q* = 0.9102 (12), θ = 94.24 (8)°, ψ = 166.77 (8)° for D, *Q* = 0.8809 (11), θ = 83.73 (8)°, ψ = 10.62 (8)° for E, and *Q* = 0.9932 (11), θ = 99.68 (7)°, ψ = 170.91 (7)° for F] is consistent with each of the metallocycles being described as having a twist-boat conformation (Cremer *et al.*, 1975 ▸). The dihedral angle between the mean planes of the two five-membered pyrazole rings found on the same bdippza ligand (*Cg*1 and *Cg*2) is 118.36°, while the dihedral angle between the mean planes of the imidazole rings *Cg*1 and *Cg*2^i^, which are on different bdippza ligands, is 61.64°.

## Supra­molecular features   

**Figure 2:**
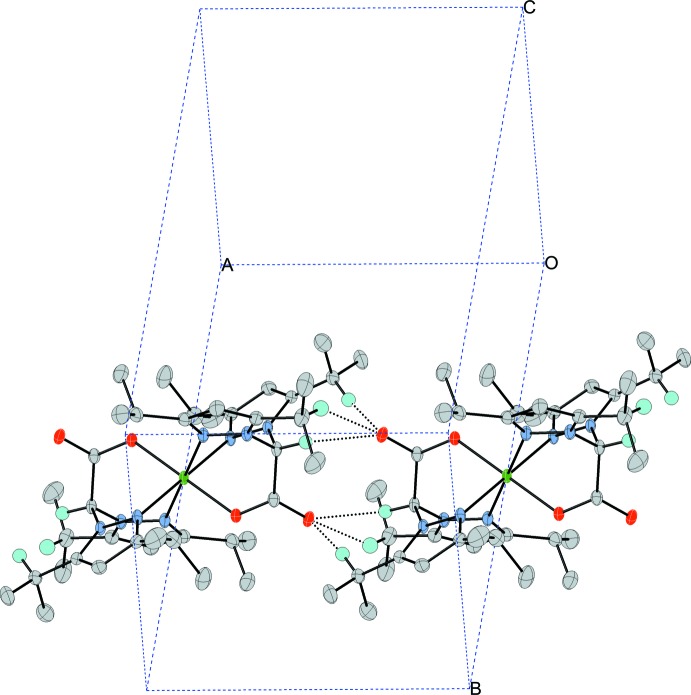
A partial unit-cell packing diagram, showing the weak C—H⋯O inter­molecular inter­actions (dashed lines). For clarity, only H atoms involved in the C—H⋯O inter­actions between adjacent mol­ecules have been included.

Within the crystal, close inter­molecular C—H⋯O contacts are present between mol­ecules, which result in the mol­ecules being packed in columns along the *a* axis. The weak C—H⋯O inter­molecular contacts consist of the carboxyl­ate oxygen (O2) at (*x*, *y*, *z*) acting as a hydrogen-bond acceptor to three C—H bonds (C4—H4, C12—H12, and C15—H15) on an adjacent complex at (−1 − *x*, −*y*, 1 − *z*), as shown in Fig. 2 ▸. Within each complex, weak π-stacking inter­actions between the imidazole rings (*Cg*1⋯*Cg*2) on the same bdippza ligand are observed. Furthermore, a weak slipped-parallel C—H⋯π (C9—H9⋯*Cg*2, *X*—H, π = 60°) inter­action is present. Full details of the hydrogen-bonding geometries and π–π inter­actions are provided in Table 2 ▸.

## Database survey   

Three related homoleptic Zn^II^ compounds containing dif­ferent substituted bis­(3,5-di­alkyl­pyrazol-1-yl)acetate sup­porting ligands [bdmpza = bis­(3,5-di­methyl­pyrazol-1-yl)acetate and bpa^tBu2,Me2^ = 3,5-di-*tert-*butyl-1-(3,5-dimethyl-1*H*-pyrazol-1-yl)acetate] have been characterized crystallographically (Pockaj *et al.*, 2015 ▸; Hegelmann *et al.*, 2003 ▸; Beck *et al.*, 2001 ▸). The Zn—O bond length in **2** [2.0472 (10) Å] is shorter compared to [(bdmpza)_2_Zn]·3H_2_O (Beck *et al.*, 2001 ▸) and [(bdmpza)_2_Zn]·2H_2_O (Pockaj *et al.*, 2015 ▸), which reported Zn—O bond lengths of 2.119 (3) and 2.100 (2) Å, respectively. The longer Zn—O bond lengths in the hydrated [(bdmpza)_2_Zn]·*x*H_2_O complexes are a consequence of O—H⋯H hydrogen-bonding inter­actions between the carboxyl­ate carbonyl O atoms and cocrystallized water mol­ecules that link adjacent coordination mol­ecules to form infinite chains. Compound **2** does not contain cocrystallized water or solvent. Conversely, the Zn—O distance in **2** is longer by 0.04 Å compared to [(bpa^tBu2,Me2^)_2_Zn] (Hegelmann *et al.*, 2003 ▸), which has a Zn—O bond length of 2.006 (3) Å. The difference in bond lengths arises from [(bpa^tBu2,Me2^)_2_Zn] having a distorted square-pyramidal environment instead of a distorted octa­hedral coordination due to one of the 3,5-di-*tert*-butyl­pyrazol-1-yl groups having a weak inter­action with the zinc ion.

## Synthesis and crystallization   

### General   

All reactions were performed using standard Schlenk techniques under a nitro­gen atmosphere. The tetra­hydro­furan (THF) solvent was distilled from sodium/benzo­phenone ketyl, while methanol was distilled from CaH_2_. NMR spectra were recorded on a Bruker AVANCE III 600 NMR. Chemical shifts are expressed in parts per million (ppm) and referenced to residual solvent as the inter­nal reference for ^1^H (CDCl_3_; δ = 7.24 ppm) and ^13^C (CDCl_3_; δ = 77.16 ppm). IR spectra were measured using a PerkinElmer Spectrum 100 spectrometer. Electrospray mass spectra were recorded on a Bruker HCTultra ETD II mass spectrometer. Bis­(3,5-diiso­propyl­pyrazol-1-yl)methane was prepared according to a previously reported procedure (Spiro­pulos *et al.*, 2011 ▸).

### Preparation of lithium bis­(3,5-diiso­propyl­pyrazol-1-yl)acetate, [Li(bdippza)] (1)   

To a solution of bis­(3,5-diiso­propyl­pyrazol-1-yl)methane (0.5 g, 1.6 mmol) dissolved in dry THF (40 ml) was added *n*BuLi (1.6 *M*, 1.5 ml, 2.4 mmol) in hexane at 195 K. After 1 h of stirring, carbon dioxide was bubbled through the solution at 233 K for 30 min. The solution then was allowed to reach ambient temperature and stirred for 2 h before the volume was reduced to 3 ml under reduced pressure. Addition of hexane (10 ml) resulted in the formation of a white solid, which was filtered off, washed with hexane (2 × 5 ml) and dried under reduced pressure (0.27 g, 47%). ^1^H NMR (CDCl_3_): δ 6.59 (*s*, 1H), 5.80 (*s*, 2H), 3.06 (heptet, *J* = 6.8 Hz, 2H), 2.83 (heptet, *J* = 6.9 Hz, 2H), 1.31 (*d*, *J* = 6.8 Hz, 6H), 1.23 (*d*, *J* = 6.8 Hz, 6H), 1.05 (*d*, *J* = 6.9 Hz, 6H), 0.98 (*d*, *J* = 6.9 Hz, 6H). FT–IR (ATR, cm^−1^): 2966 (*m*), 2930 (*m*), 2870 (*m*), 1676 (*m*), 1643 (*s*), 1551 (*m*), 1458 (*m*), 1408 (*m*), 1373 (*m*), 1310 (*m*), 1284 (*m*), 1226 (*m*), 1181 (*m*), 1104 (*m*), 1073 (*m*), 1060 (*m*), 1012 (*m*), 912 (*m*), 861 (*m*), 792 (*s*), 771 (*m*), 738 (*m*), 723 (*m*), 686 (*m*). MS (ESI, neg): *m*/*z* found for [C_20_H_31_N_4_O_2_ −Li]^−^, 359; [C_19_H_31_N_4_ − Li − CO_2_]^−^, 315.

### Preparation of [(bdippza)_2_Zn]   

ZnCl_2_ (0.015 g, 0.11 mmol) was added to [Li(bdippza)] (**1**) (0.083 g, 0.23 mmol) in dry MeOH (15 ml). The reaction was stirred for 24 h, during which time a white solid formed. The solvent was removed under reduced pressure, di­chloro­methane (15 ml) was added, and the solution filtered through celite. The volume was reduced (∼3 ml) and addition of hexane (10 ml) caused the formation of a white solid. The solid was collected, washed with hexane (2 × 5 ml), and dried under vacuum (0.069 g, 78%). Colorless crystals suitable for crystallographic characterization were obtained by hexane diffusion into THF at room temperature. ^1^H NMR (CDCl_3_): δ 6.56 (*s*, 2H, CH), 6.00 (*s*, 4H, H_pz_), 3.59–3.47 (*m*, 4H, CH-^i^Pr), 3.02 (heptet, *J* = 6.8 Hz, 4H, CH-^i^Pr), 1.37 (*d*, *J* = 6.8 Hz, 12H, CH_3_-^i^Pr), 1.30 (*d*, *J* = 6.8 Hz, 12H, CH_3_-^i^Pr), 1.19 (*d*, *J* = 6.9 Hz, 12H, CH_3_-^i^Pr), 1.02 (*d*, *J* = 6.9 Hz, 12H, CH_3_-^i^Pr). ^13^C NMR (CDCl_3_): δ 165.8 (CO_2_^−^), 163.9 (C_pz_), 154.6 (C_pz_), 99.6 (C_pz_), 67.0 (CH), 27.2 (CH-^i^Pr), 25.9 (CH-^i^Pr), 23.3(CH_3_-^i^Pr), 22.8 (CH_3_-^i^Pr), 22.4 (CH_3_-^i^Pr), 22.1 (CH_3_-^i^Pr). FT–IR (ATR, cm^−1^): 2966 (*m*), 2932 (*m*), 2871 (*m*), 1687 (*s*, CO_2_^−^), 1552 (*m*, C=N), 1475 (*m*), 1460 (*m*), 1409 (*m*), 1356 (*s*), 1315 (*m*), 1292 (*m*), 1252 (*m*), 1184 (*m*), 1088 (*m*), 1059 (*m*), 1024 (*m*), 910 (*m*), 854 (*m*), 798 (*s*), 778 (*s*), 724 (*m*), 692 (*s*). MS (ESI, pos): *m*/*z* found for [C_40_H_62_N_8_O_4_Zn + Na]^+^, 805.

## Refinement   

Crystal data, data collection and structure refinement details are summarized in Table 3 ▸.

## Supplementary Material

Crystal structure: contains datablock(s) I. DOI: 10.1107/S2056989018011246/jj2201sup1.cif

Structure factors: contains datablock(s) I. DOI: 10.1107/S2056989018011246/jj2201Isup2.hkl

NMR (1H, 13C{1H}, HMBC, HSQC and COSY) and ESI-MS spectra for the titled compound are provided.. DOI: 10.1107/S2056989018011246/jj2201sup3.pdf

CCDC reference: 1860544

Additional supporting information:  crystallographic information; 3D view; checkCIF report

## Figures and Tables

**Table 1:** Selected geometric parameters (Å, °)

Zn1—O1	2.0472 (10)	Zn1—N1	2.1941 (12)
Zn1—N4	2.1674 (11)		
			
O1—Zn1—N4	86.40 (4)	N4—Zn1—N1	82.91 (4)
O1—Zn1—N4^i^	93.60 (4)	O1—Zn1—N1^i^	93.49 (4)
O1—Zn1—N1	86.51 (4)	N4—Zn1—N1^i^	97.09 (4)

Symmetry code: (i) 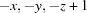.

**Table 2:** Hydrogen-bonding geometry and π–π inter­actions (Å, °) *Cg*1 and *Cg*2 are the centroids of the N1/N2/C3/C2/C1 and N3/N4/C7/C6/C5 rings, respectively.

*D*—H⋯*A*	*D*—H	H⋯*A*	*D*⋯*A*	*D*—H⋯*A*
C4—H4⋯O2^i^	0.95	2.44	3.3883 (18)	172
C12—H12⋯O2^i^	0.94	2.34	3.223 (2)	156
C15—H15⋯O2^i^	0.94	2.44	3.229 (2)	141
*Cg*1⋯*Cg*2			4.2001 (9)	
C9—H9⋯*Cg*2	0.99	2.97	3.9410 (18)	168

Symmetry code: (i) −*x* − 1, −*y*, −*z* + 1.

**Table 3:** Experimental details

Crystal data	
Chemical formula	[Zn(C_20_H_31_N_4_O_2_)_2_]
*M*_r_	784.35
Crystal system, space group	Monoclinic, *P*2_1_/*c*
Temperature (K)	150
*a*, *b*, *c* (Å)	10.1806 (1), 16.9578 (3), 12.4534 (2)
β (°)	96.9735 (10)
*V* (Å^3^)	2134.06 (6)
*Z*	2
Radiation type	Mo *K*α
μ (mm^−1^)	0.62
Crystal size (mm)	0.25 × 0.18 × 0.13

Data collection	
Diffractometer	Nonius KappaCCD
Absorption correction	Multi-scan (*DENZO-SMN*; Otwinowski & Minor, 1997 ▸)
*T*_min_, *T*_max_	0.860, 0.923
No. of measured, independent and observed [*I* > 2σ(*I*)] reflections	9825, 5072, 3881
*R*_int_	0.033
(sin θ/λ)_max_ (Å^−1^)	0.658

Refinement	
*R*[*F* ^2^ > 2σ(*F* ^2^)], *wR*(*F* ^2^), *S*	0.033, 0.075, 1.03
No. of reflections	5072
No. of parameters	365
H-atom treatment	All H-atom parameters refined
Δρ_max_, Δρ_min_ (e Å^−3^)	0.28, −0.41

Computer programs: *COLLECT* (Nonius, 1998 ▸), *DENZO-SMN* (Otwinowski & Minor, 1997 ▸), *SIR97* (Altomare *et al.*, 1999 ▸), *SHELXL97* (Sheldrick, 2008 ▸) and *WinGX* and *ORTEP-3* (Farrugia, 2012 ▸).

## supplementary crystallographic information

### Crystal data

**Table:**

[Zn(C_20_H_31_N_4_O_2_)_2_]	*F*(000) = 840
*M**_r_* = 784.35	*D*_x_ = 1.221 Mg m^−^^3^
Monoclinic, *P*2_1_/*c*	Mo *K*α radiation, λ = 0.71073 Å
Hall symbol: -P 2ybc	Cell parameters from 8352 reflections
*a* = 10.1806 (1) Å	θ = 1.0–20.4°
*b* = 16.9578 (3) Å	µ = 0.62 mm^−^^1^
*c* = 12.4534 (2) Å	*T* = 150 K
β = 96.9735 (10)°	Prism, colorless
*V* = 2134.06 (6) Å^3^	0.25 × 0.18 × 0.13 mm
*Z* = 2	

### Data collection

**Table:**

Nonius KappaCCD diffractometer	5072 independent reflections
Radiation source: fine-focus sealed tube	3881 reflections with *I* > 2σ(*I*)
Graphite monochromator	*R*_int_ = 0.033
Phi and ω scan	θ_max_ = 27.9°, θ_min_ = 2.4°
Absorption correction: multi-scan (DENZO-SMN; Otwinowski & Minor, 1997)	*h* = −13→13
*T*_min_ = 0.860, *T*_max_ = 0.923	*k* = −22→22
9825 measured reflections	*l* = −16→16

### Refinement

**Table:**

Refinement on *F*^2^	Primary atom site location: structure-invariant direct methods
Least-squares matrix: full	Secondary atom site location: difference Fourier map
*R*[*F*^2^ > 2σ(*F*^2^)] = 0.033	Hydrogen site location: inferred from neighbouring sites
*wR*(*F*^2^) = 0.075	All H-atom parameters refined
*S* = 1.03	*w* = 1/[σ^2^(*F*_o_^2^) + (0.0287*P*)^2^ + 0.6212*P*] where *P* = (*F*_o_^2^ + 2*F*_c_^2^)/3
5072 reflections	(Δ/σ)_max_ < 0.001
365 parameters	Δρ_max_ = 0.28 e Å^−^^3^
0 restraints	Δρ_min_ = −0.41 e Å^−^^3^

### Special details

**Table:**

Experimental. The program Denzo-SMN (Otwinowski & Minor, 1997) uses a scaling algorithm (Fox & Holmes, 1966) which effectively corrects for absorption effects. High redundancy data were used in the scaling program hence the 'multi-scan' code word was used. No transmission coefficients are available from the program (only scale factors for each frame). The scale factors in the experimental table are calculated from the 'size' command in the SHELXL-97 input file.
Geometry. All esds (except the esd in the dihedral angle between two l.s. planes) are estimated using the full covariance matrix. The cell esds are taken into account individually in the estimation of esds in distances, angles and torsion angles; correlations between esds in cell parameters are only used when they are defined by crystal symmetry. An approximate (isotropic) treatment of cell esds is used for estimating esds involving l.s. planes.
Refinement. Refinement of F^2^ against ALL reflections. The weighted R-factor wR and goodness of fit S are based on F^2^, conventional R-factors R are based on F, with F set to zero for negative F^2^. The threshold expression of F^2^ > 2sigma(F^2^) is used only for calculating R-factors(gt) etc. and is not relevant to the choice of reflections for refinement. R-factors based on F^2^ are statistically about twice as large as those based on F, and R- factors based on ALL data will be even larger.

### Fractional atomic coordinates and isotropic or equivalent isotropic displacement parameters (Å^2^)

**Table:**

	*x*	*y*	*z*	*U*_iso_*/*U*_eq_	
Zn1	0.0000	0.0000	0.5000	0.01725 (8)	
O1	−0.17165 (10)	−0.05936 (6)	0.44996 (8)	0.0209 (2)	
O2	−0.39140 (10)	−0.04825 (7)	0.41634 (9)	0.0265 (3)	
N1	−0.11682 (11)	0.10849 (7)	0.47319 (9)	0.0176 (3)	
N2	−0.23516 (11)	0.10762 (7)	0.51632 (9)	0.0163 (3)	
N3	−0.20337 (11)	0.00927 (7)	0.65508 (9)	0.0173 (3)	
N4	−0.06882 (11)	0.00496 (7)	0.65787 (9)	0.0182 (3)	
C1	−0.10013 (14)	0.18293 (9)	0.44361 (12)	0.0202 (3)	
C2	−0.20742 (15)	0.22944 (9)	0.46651 (13)	0.0230 (3)	
C3	−0.29170 (14)	0.18053 (9)	0.51367 (12)	0.0184 (3)	
C4	−0.28291 (14)	0.03319 (9)	0.55466 (11)	0.0163 (3)	
C5	−0.24317 (15)	−0.01243 (9)	0.75104 (11)	0.0201 (3)	
C6	−0.12943 (16)	−0.02940 (10)	0.81833 (12)	0.0243 (3)	
C7	−0.02332 (15)	−0.01781 (9)	0.75795 (12)	0.0201 (3)	
C8	−0.28289 (14)	−0.03104 (9)	0.46471 (11)	0.0168 (3)	
C9	0.02168 (16)	0.20845 (10)	0.39572 (14)	0.0283 (4)	
C10	−0.0141 (2)	0.26302 (13)	0.29895 (17)	0.0421 (5)	
C11	0.11899 (19)	0.24866 (13)	0.48170 (18)	0.0388 (5)	
C12	−0.41863 (15)	0.19758 (10)	0.55931 (13)	0.0243 (3)	
C13	−0.48545 (18)	0.27096 (11)	0.50744 (18)	0.0360 (4)	
C14	−0.3943 (2)	0.20583 (13)	0.68232 (15)	0.0377 (4)	
C15	−0.38671 (16)	−0.02061 (11)	0.76703 (13)	0.0267 (4)	
C16	−0.4041 (2)	−0.01540 (17)	0.88672 (15)	0.0437 (6)	
C17	−0.4420 (2)	−0.09813 (14)	0.71835 (18)	0.0435 (5)	
C18	0.12297 (15)	−0.02653 (10)	0.79192 (13)	0.0245 (3)	
C19	0.18716 (18)	0.05324 (12)	0.81940 (16)	0.0328 (4)	
C20	0.1505 (2)	−0.08334 (12)	0.88676 (16)	0.0361 (4)	
H2	−0.2196 (16)	0.2832 (10)	0.4523 (13)	0.024 (4)*	
H4	−0.3720 (16)	0.0402 (9)	0.5694 (12)	0.018 (4)*	
H6	−0.1259 (16)	−0.0462 (10)	0.8923 (14)	0.027 (4)*	
H9	0.0639 (17)	0.1606 (11)	0.3703 (14)	0.031 (5)*	
H10A	−0.079 (2)	0.2389 (13)	0.2427 (18)	0.055 (6)*	
H10B	0.064 (2)	0.2722 (12)	0.2667 (17)	0.052 (6)*	
H10C	−0.0515 (19)	0.3126 (12)	0.3223 (16)	0.043 (6)*	
H11A	0.1456 (19)	0.2147 (12)	0.5427 (16)	0.041 (5)*	
H11B	0.197 (2)	0.2639 (12)	0.4482 (16)	0.046 (6)*	
H11C	0.0797 (19)	0.2952 (11)	0.5102 (15)	0.036 (5)*	
H12	−0.4765 (16)	0.1549 (10)	0.5438 (13)	0.019 (4)*	
H13A	−0.568 (2)	0.2786 (11)	0.5388 (15)	0.044 (6)*	
H13B	−0.504 (2)	0.2641 (13)	0.4277 (18)	0.052 (6)*	
H13C	−0.429 (2)	0.3190 (12)	0.5261 (15)	0.044 (5)*	
H14A	−0.479 (2)	0.2095 (13)	0.7118 (17)	0.059 (6)*	
H14B	−0.344 (2)	0.1589 (12)	0.7184 (15)	0.043 (5)*	
H14C	−0.342 (2)	0.2497 (12)	0.7026 (15)	0.039 (5)*	
H15	−0.4339 (17)	0.0219 (10)	0.7318 (14)	0.024 (4)*	
H16A	−0.498 (2)	−0.0199 (13)	0.8966 (17)	0.054 (6)*	
H16C	−0.3739 (19)	0.0372 (12)	0.9114 (15)	0.036 (5)*	
H16B	−0.358 (2)	−0.0561 (13)	0.9265 (17)	0.052 (6)*	
H17A	−0.432 (2)	−0.0998 (12)	0.6390 (17)	0.051 (6)*	
H17B	−0.392 (2)	−0.1428 (12)	0.7557 (16)	0.047 (6)*	
H17C	−0.536 (2)	−0.1047 (13)	0.7273 (17)	0.055 (6)*	
H18	0.1625 (16)	−0.0474 (10)	0.7325 (13)	0.023 (4)*	
H19A	0.1506 (17)	0.0783 (10)	0.8825 (15)	0.032 (5)*	
H19B	0.285 (2)	0.0461 (12)	0.8343 (16)	0.050 (6)*	
H19C	0.1748 (18)	0.0875 (11)	0.7587 (16)	0.036 (5)*	
H20A	0.107 (2)	−0.1343 (13)	0.8722 (16)	0.046 (6)*	
H20B	0.117 (2)	−0.0619 (12)	0.9539 (17)	0.049 (6)*	
H20C	0.240 (2)	−0.0920 (12)	0.9050 (16)	0.048 (6)*	

### Atomic displacement parameters (Å^2^)

**Table:**

	*U*^11^	*U*^22^	*U*^33^	*U*^12^	*U*^13^	*U*^23^
Zn1	0.01007 (11)	0.02354 (14)	0.01834 (12)	0.00096 (10)	0.00251 (9)	−0.00057 (10)
O1	0.0121 (5)	0.0256 (6)	0.0249 (5)	0.0003 (4)	0.0022 (4)	−0.0045 (4)
O2	0.0128 (5)	0.0374 (7)	0.0286 (6)	−0.0035 (5)	0.0005 (5)	−0.0088 (5)
N1	0.0097 (6)	0.0228 (7)	0.0211 (6)	−0.0002 (5)	0.0050 (5)	0.0009 (5)
N2	0.0094 (5)	0.0213 (6)	0.0185 (6)	−0.0001 (5)	0.0031 (5)	−0.0006 (5)
N3	0.0120 (5)	0.0257 (7)	0.0143 (6)	0.0006 (5)	0.0026 (5)	0.0012 (5)
N4	0.0116 (5)	0.0247 (7)	0.0181 (6)	0.0016 (5)	0.0007 (5)	−0.0001 (5)
C1	0.0169 (7)	0.0218 (8)	0.0220 (8)	−0.0037 (6)	0.0027 (6)	−0.0032 (6)
C2	0.0221 (8)	0.0167 (8)	0.0305 (9)	−0.0017 (6)	0.0045 (7)	−0.0029 (6)
C3	0.0141 (7)	0.0205 (8)	0.0198 (7)	0.0004 (6)	−0.0005 (6)	−0.0051 (6)
C4	0.0098 (6)	0.0223 (8)	0.0171 (7)	−0.0004 (6)	0.0027 (6)	0.0023 (6)
C5	0.0194 (7)	0.0265 (9)	0.0148 (7)	−0.0006 (6)	0.0041 (6)	−0.0014 (6)
C6	0.0248 (8)	0.0330 (9)	0.0149 (7)	0.0014 (7)	0.0011 (6)	0.0006 (6)
C7	0.0193 (7)	0.0221 (8)	0.0182 (7)	0.0035 (6)	−0.0005 (6)	−0.0021 (6)
C8	0.0143 (7)	0.0201 (7)	0.0166 (7)	−0.0018 (6)	0.0042 (6)	0.0038 (6)
C9	0.0226 (8)	0.0247 (9)	0.0400 (10)	−0.0060 (7)	0.0139 (8)	0.0004 (7)
C10	0.0447 (12)	0.0462 (13)	0.0372 (11)	−0.0198 (10)	0.0127 (10)	0.0036 (9)
C11	0.0227 (9)	0.0415 (12)	0.0518 (13)	−0.0100 (9)	0.0031 (9)	0.0056 (10)
C12	0.0159 (7)	0.0238 (8)	0.0341 (9)	−0.0004 (7)	0.0064 (7)	−0.0072 (7)
C13	0.0219 (9)	0.0324 (10)	0.0535 (13)	0.0079 (8)	0.0041 (9)	−0.0048 (9)
C14	0.0342 (10)	0.0455 (12)	0.0352 (10)	0.0055 (10)	0.0110 (9)	−0.0117 (9)
C15	0.0202 (8)	0.0436 (11)	0.0174 (7)	−0.0039 (7)	0.0064 (7)	0.0024 (7)
C16	0.0268 (9)	0.0835 (19)	0.0223 (9)	−0.0041 (11)	0.0093 (8)	0.0009 (10)
C17	0.0381 (11)	0.0528 (14)	0.0402 (12)	−0.0201 (10)	0.0079 (10)	0.0006 (10)
C18	0.0205 (8)	0.0310 (9)	0.0206 (8)	0.0072 (7)	−0.0031 (7)	−0.0043 (6)
C19	0.0247 (9)	0.0362 (10)	0.0350 (10)	0.0004 (8)	−0.0066 (8)	−0.0023 (8)
C20	0.0341 (10)	0.0375 (11)	0.0330 (10)	0.0086 (9)	−0.0110 (9)	0.0033 (8)

### Geometric parameters (Å, º)

**Table:**

Zn1—O1^i^	2.0471 (10)	C10—H10B	0.94 (2)
Zn1—O1	2.0472 (10)	C10—H10C	0.98 (2)
Zn1—N4	2.1674 (11)	C11—H11A	0.96 (2)
Zn1—N4^i^	2.1675 (11)	C11—H11B	0.98 (2)
Zn1—N1	2.1941 (12)	C11—H11C	0.971 (19)
Zn1—N1^i^	2.1942 (12)	C12—C13	1.524 (2)
O1—C8	1.2640 (17)	C12—C14	1.528 (2)
O2—C8	1.2275 (17)	C12—H12	0.938 (17)
N1—C1	1.3317 (19)	C13—H13A	0.98 (2)
N1—N2	1.3774 (15)	C13—H13B	0.99 (2)
N2—C3	1.3625 (19)	C13—H13C	1.01 (2)
N2—C4	1.4539 (18)	C14—H14A	0.98 (2)
N3—C5	1.3583 (19)	C14—H14B	1.02 (2)
N3—N4	1.3680 (15)	C14—H14C	0.93 (2)
N3—C4	1.4623 (18)	C15—C16	1.525 (2)
N4—C7	1.3330 (19)	C15—C17	1.526 (3)
C1—C2	1.404 (2)	C15—H15	0.945 (18)
C1—C9	1.503 (2)	C16—H16A	0.98 (2)
C2—C3	1.375 (2)	C16—H16C	0.98 (2)
C2—H2	0.933 (17)	C16—H16B	0.94 (2)
C3—C12	1.5016 (19)	C17—H17A	1.01 (2)
C4—C8	1.562 (2)	C17—H17B	1.00 (2)
C4—H4	0.954 (16)	C17—H17C	0.98 (2)
C5—C6	1.375 (2)	C18—C19	1.523 (2)
C5—C15	1.505 (2)	C18—C20	1.524 (2)
C6—C7	1.403 (2)	C18—H18	0.953 (16)
C6—H6	0.960 (17)	C19—H19A	1.004 (18)
C7—C18	1.505 (2)	C19—H19B	1.00 (2)
C9—C10	1.528 (3)	C19—H19C	0.949 (19)
C9—C11	1.528 (3)	C20—H20A	0.98 (2)
C9—H9	0.988 (18)	C20—H20B	1.01 (2)
C10—H10A	0.99 (2)	C20—H20C	0.93 (2)
			
O1^i^—Zn1—O1	180.0	C9—C10—H10C	110.5 (12)
O1^i^—Zn1—N4	93.60 (4)	H10A—C10—H10C	108.5 (18)
O1—Zn1—N4	86.40 (4)	H10B—C10—H10C	111.1 (17)
O1^i^—Zn1—N4^i^	86.40 (4)	C9—C11—H11A	112.4 (12)
O1—Zn1—N4^i^	93.60 (4)	C9—C11—H11B	108.1 (12)
N4—Zn1—N4^i^	180.0	H11A—C11—H11B	109.2 (16)
O1^i^—Zn1—N1	93.49 (4)	C9—C11—H11C	111.0 (12)
O1—Zn1—N1	86.51 (4)	H11A—C11—H11C	106.6 (16)
N4—Zn1—N1	82.91 (4)	H11B—C11—H11C	109.5 (16)
N4^i^—Zn1—N1	97.09 (4)	C3—C12—C13	110.93 (14)
O1^i^—Zn1—N1^i^	86.51 (4)	C3—C12—C14	110.77 (14)
O1—Zn1—N1^i^	93.49 (4)	C13—C12—C14	111.10 (15)
N4—Zn1—N1^i^	97.09 (4)	C3—C12—H12	108.8 (9)
N4^i^—Zn1—N1^i^	82.91 (4)	C13—C12—H12	107.8 (10)
N1—Zn1—N1^i^	180.00 (3)	C14—C12—H12	107.4 (10)
C8—O1—Zn1	121.05 (9)	C12—C13—H13A	107.5 (12)
C1—N1—N2	105.37 (11)	C12—C13—H13B	110.4 (12)
C1—N1—Zn1	138.76 (10)	H13A—C13—H13B	110.0 (17)
N2—N1—Zn1	114.54 (9)	C12—C13—H13C	110.5 (12)
C3—N2—N1	111.58 (11)	H13A—C13—H13C	107.2 (15)
C3—N2—C4	129.68 (11)	H13B—C13—H13C	111.1 (16)
N1—N2—C4	118.72 (11)	C12—C14—H14A	109.7 (13)
C5—N3—N4	111.59 (11)	C12—C14—H14B	112.4 (11)
C5—N3—C4	129.36 (12)	H14A—C14—H14B	107.7 (16)
N4—N3—C4	119.03 (11)	C12—C14—H14C	111.3 (12)
C7—N4—N3	105.81 (11)	H14A—C14—H14C	110.2 (17)
C7—N4—Zn1	136.30 (10)	H14B—C14—H14C	105.3 (16)
N3—N4—Zn1	114.33 (8)	C5—C15—C16	110.73 (14)
N1—C1—C2	110.35 (13)	C5—C15—C17	110.12 (15)
N1—C1—C9	121.44 (13)	C16—C15—C17	110.92 (16)
C2—C1—C9	128.20 (14)	C5—C15—H15	108.4 (10)
C3—C2—C1	106.80 (14)	C16—C15—H15	107.3 (10)
C3—C2—H2	126.4 (10)	C17—C15—H15	109.3 (10)
C1—C2—H2	126.8 (10)	C15—C16—H16A	110.4 (13)
N2—C3—C2	105.90 (12)	C15—C16—H16C	106.8 (11)
N2—C3—C12	123.10 (13)	H16A—C16—H16C	107.8 (16)
C2—C3—C12	130.97 (14)	C15—C16—H16B	111.2 (13)
N2—C4—N3	110.44 (11)	H16A—C16—H16B	108.0 (18)
N2—C4—C8	109.91 (11)	H16C—C16—H16B	112.5 (18)
N3—C4—C8	111.81 (12)	C15—C17—H17A	109.8 (12)
N2—C4—H4	108.6 (10)	C15—C17—H17B	108.9 (12)
N3—C4—H4	108.1 (9)	H17A—C17—H17B	109.3 (16)
C8—C4—H4	107.9 (10)	C15—C17—H17C	111.7 (13)
N3—C5—C6	105.89 (13)	H17A—C17—H17C	108.7 (17)
N3—C5—C15	122.65 (13)	H17B—C17—H17C	108.4 (17)
C6—C5—C15	131.26 (14)	C7—C18—C19	111.02 (14)
C5—C6—C7	106.87 (13)	C7—C18—C20	111.26 (14)
C5—C6—H6	125.2 (10)	C19—C18—C20	110.72 (14)
C7—C6—H6	127.9 (10)	C7—C18—H18	108.4 (10)
N4—C7—C6	109.81 (13)	C19—C18—H18	107.1 (10)
N4—C7—C18	120.72 (13)	C20—C18—H18	108.2 (10)
C6—C7—C18	129.46 (14)	C18—C19—H19A	111.2 (10)
O2—C8—O1	127.38 (14)	C18—C19—H19B	109.0 (12)
O2—C8—C4	116.06 (12)	H19A—C19—H19B	111.2 (15)
O1—C8—C4	116.56 (12)	C18—C19—H19C	110.6 (11)
C1—C9—C10	110.96 (15)	H19A—C19—H19C	109.8 (15)
C1—C9—C11	110.26 (14)	H19B—C19—H19C	104.8 (16)
C10—C9—C11	110.77 (15)	C18—C20—H20A	112.2 (12)
C1—C9—H9	107.6 (10)	C18—C20—H20B	111.5 (12)
C10—C9—H9	108.5 (10)	H20A—C20—H20B	106.3 (16)
C11—C9—H9	108.7 (10)	C18—C20—H20C	111.9 (13)
C9—C10—H10A	112.5 (13)	H20A—C20—H20C	108.7 (17)
C9—C10—H10B	107.7 (13)	H20B—C20—H20C	105.9 (17)
H10A—C10—H10B	106.6 (17)		
			
O1^i^—Zn1—O1—C8	16 (15)	C1—C2—C3—C12	177.01 (15)
N4—Zn1—O1—C8	53.53 (11)	C3—N2—C4—N3	−109.26 (15)
N4^i^—Zn1—O1—C8	−126.47 (11)	N1—N2—C4—N3	72.21 (15)
N1—Zn1—O1—C8	−29.57 (11)	C3—N2—C4—C8	126.92 (15)
N1^i^—Zn1—O1—C8	150.43 (11)	N1—N2—C4—C8	−51.62 (16)
O1^i^—Zn1—N1—C1	31.57 (15)	C5—N3—C4—N2	128.41 (15)
O1—Zn1—N1—C1	−148.43 (15)	N4—N3—C4—N2	−53.40 (16)
N4—Zn1—N1—C1	124.77 (15)	C5—N3—C4—C8	−108.87 (16)
N4^i^—Zn1—N1—C1	−55.23 (15)	N4—N3—C4—C8	69.32 (15)
N1^i^—Zn1—N1—C1	−92 (11)	N4—N3—C5—C6	1.46 (17)
O1^i^—Zn1—N1—N2	−132.72 (9)	C4—N3—C5—C6	179.76 (14)
O1—Zn1—N1—N2	47.28 (9)	N4—N3—C5—C15	−173.97 (13)
N4—Zn1—N1—N2	−39.52 (9)	C4—N3—C5—C15	4.3 (2)
N4^i^—Zn1—N1—N2	140.48 (9)	N3—C5—C6—C7	−0.73 (17)
N1^i^—Zn1—N1—N2	104 (11)	C15—C5—C6—C7	174.15 (16)
C1—N1—N2—C3	0.03 (15)	N3—N4—C7—C6	1.08 (16)
Zn1—N1—N2—C3	169.36 (9)	Zn1—N4—C7—C6	−155.28 (12)
C1—N1—N2—C4	178.82 (12)	N3—N4—C7—C18	−178.12 (13)
Zn1—N1—N2—C4	−11.85 (15)	Zn1—N4—C7—C18	25.5 (2)
C5—N3—N4—C7	−1.60 (16)	C5—C6—C7—N4	−0.23 (18)
C4—N3—N4—C7	179.91 (12)	C5—C6—C7—C18	178.88 (15)
C5—N3—N4—Zn1	160.70 (10)	Zn1—O1—C8—O2	159.40 (12)
C4—N3—N4—Zn1	−17.80 (15)	Zn1—O1—C8—C4	−20.61 (16)
O1^i^—Zn1—N4—C7	−57.11 (15)	N2—C4—C8—O2	−104.57 (14)
O1—Zn1—N4—C7	122.89 (15)	N3—C4—C8—O2	132.40 (13)
N4^i^—Zn1—N4—C7	−160 (6)	N2—C4—C8—O1	75.44 (16)
N1—Zn1—N4—C7	−150.19 (15)	N3—C4—C8—O1	−47.58 (16)
N1^i^—Zn1—N4—C7	29.81 (15)	N1—C1—C9—C10	136.65 (16)
O1^i^—Zn1—N4—N3	147.94 (9)	C2—C1—C9—C10	−45.1 (2)
O1—Zn1—N4—N3	−32.06 (9)	N1—C1—C9—C11	−100.24 (18)
N4^i^—Zn1—N4—N3	45 (6)	C2—C1—C9—C11	78.0 (2)
N1—Zn1—N4—N3	54.86 (9)	N2—C3—C12—C13	−157.28 (15)
N1^i^—Zn1—N4—N3	−125.13 (9)	C2—C3—C12—C13	25.2 (2)
N2—N1—C1—C2	−0.58 (16)	N2—C3—C12—C14	78.85 (19)
Zn1—N1—C1—C2	−165.78 (11)	C2—C3—C12—C14	−98.7 (2)
N2—N1—C1—C9	177.97 (13)	N3—C5—C15—C16	−159.12 (17)
Zn1—N1—C1—C9	12.8 (2)	C6—C5—C15—C16	26.7 (3)
N1—C1—C2—C3	0.91 (18)	N3—C5—C15—C17	77.8 (2)
C9—C1—C2—C3	−177.51 (15)	C6—C5—C15—C17	−96.3 (2)
N1—N2—C3—C2	0.52 (16)	N4—C7—C18—C19	79.98 (18)
C4—N2—C3—C2	−178.10 (13)	C6—C7—C18—C19	−99.0 (2)
N1—N2—C3—C12	−177.54 (13)	N4—C7—C18—C20	−156.22 (15)
C4—N2—C3—C12	3.8 (2)	C6—C7—C18—C20	24.8 (2)
C1—C2—C3—N2	−0.84 (16)		

Symmetry code: (i) −*x*, −*y*, −*z*+1.

## References

[bb1] Altomare, A., Burla, M. C., Camalli, M., Cascarano, G. L., Giacovazzo, C., Guagliardi, A., Moliterni, A. G. G., Polidori, G. & Spagna, R. (1999). *J. Appl. Cryst.* **32**, 115–119.

[bb2] Beck, A., Weibert, B. & Burzlaff, N. (2001). *Eur. J. Inorg. Chem.* pp. 521–527.

[bb3] Cremer, D. & Pople, J. A. (1975). *J. Am. Chem. Soc.* **97**, 1354–1358.

[bb4] Farrugia, L. J. (2012). *J. Appl. Cryst.* **45**, 849–854.

[bb5] Hegelmann, I., Beck, A., Eichhorn, C., Weibert, B. & Burzlaff, N. (2003). *Eur. J. Inorg. Chem.* pp. 339–347.

[bb6] Heinrikson, R. L. (1977). *Methods Enzymol.* **47**, 175–189. 10.1016/0076-6879(77)47022-8 927174

[bb7] Holland, D. R., Hausrath, A. C., Juers, D. & Matthews, B. W. (1995). *Protein Sci.* **4**, 1955–1965. 10.1002/pro.5560041001 PMC2142975 8535232

[bb8] Lipscomb, W. N. (1970). *Acc. Chem. Res.* **3**, 81–89.

[bb9] Nonius (1998). *COLLECT*. Nonius BV, Delft, The Netherlands.

[bb10] Otwinowski, Z. & Minor, W. (1997). *Methods in Enzymology*, Vol. 276, *Macromolecular Crystallography*, Part A, edited by C. W. Carter Jr & R. M. Sweet, pp. 307–326. New York: Academic Press. 10.1016/S0076-6879(97)76066-X 27754618

[bb11] Pockaj, M., Kozlevcar, B. & Kitanovski, N. (2015). *Acta Chim. Slov.* **62**, 272–280. 10.17344/acsi.2014.1048 26085407

[bb12] Rees, D. C., Lewis, M. & Lipscomb, W. N. (1983). *J. Mol. Biol.* **168**, 367–387. 10.1016/s0022-2836(83)80024-2 6887246

[bb13] Sheldrick, G. M. (2008). *Acta Cryst.* A**64**, 112–122. 10.1107/S0108767307043930 18156677

[bb14] Spiropulos, N. G., Chingas, G. C., Sullivan, M., York, J. T. & Brown, E. C. (2011). *Inorg. Chim. Acta*, **376**, 562–573.

