# Supplementary material for: Crystal structure of a homoleptic zinc(II) complex based on bis­(3,5-diiso­propyl­pyrazol-1-yl)acetate
Source: Acta Crystallogr E Crystallogr Commun. 2018 Aug 16;74(Pt 9):1259–62. doi: 10.1107/S2056989018011246 (PMC6127694; doi:10.1107/S2056989018011246)
Supplement: Supplementary file 3 [file e-74-01259-sup3.pdf]

Supporting Information

for

Crystal Structure of the Homoleptic Zinc(II) Complex Based on *bis*(3,5-Diisopropylpyrazol-1-yl)acetate

Josiah G. Elsberg, Nicholas G. Spiropulos, Adam C. Colson, Eric C. Brown*

†Department of Chemistry, Boise State University, Boise, ID 83725

Tel: 208-426-1186

Fax: 208-426-1311

E-mail: *ericbrown3@boisestate.edu*

*To whom all correspondence should be addressed

Contents

Figure S1. ^{1}H NMR spectrum of **2**……………………………………………………….…..…..S2

Figure S2. $^{13}C\{^{1}H\}$ NMR spectrum of **2**…..……………………….……………………..…..S2

Figure S3. HMBC NMR spectrum of **2**………..……………………….………….……….…..…..S3

Figure S4. HSQC NMR spectrum of **2**…………………………………………………...…S3

Figure S5 COSY NMR spectrum of **2**……………………………………………………………S4

Figure S6. ESI Mass spectrometry of **2**….…………………………….…………………….....S4

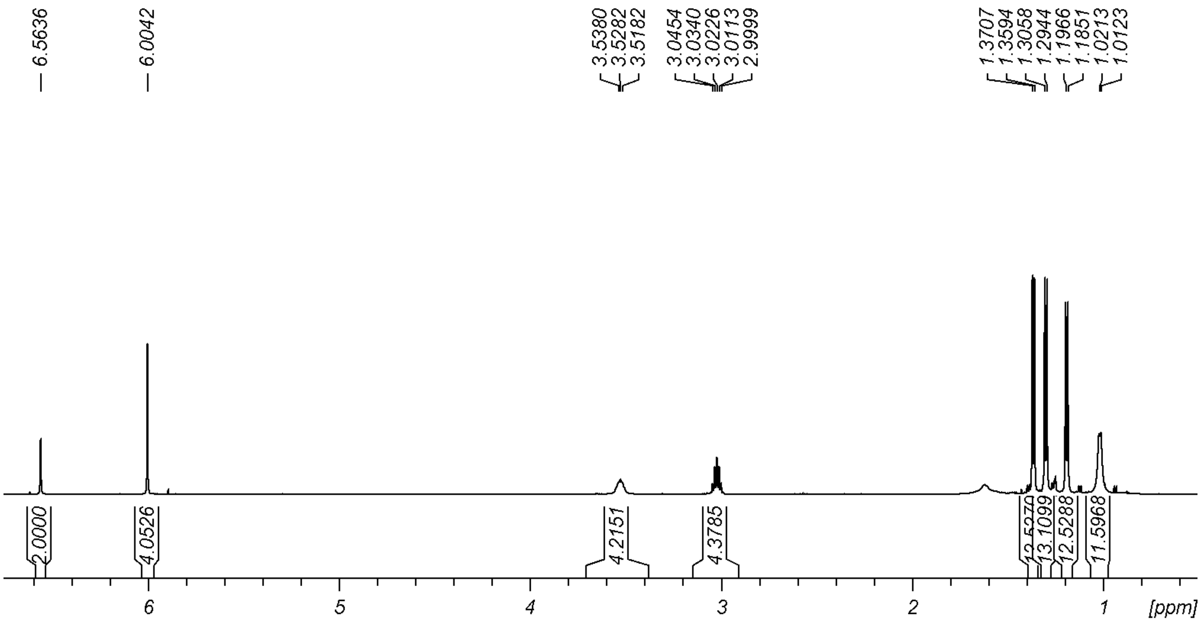

Figure S1. ^{1}H NMR spectrum of **2** in $CDCl_3$.

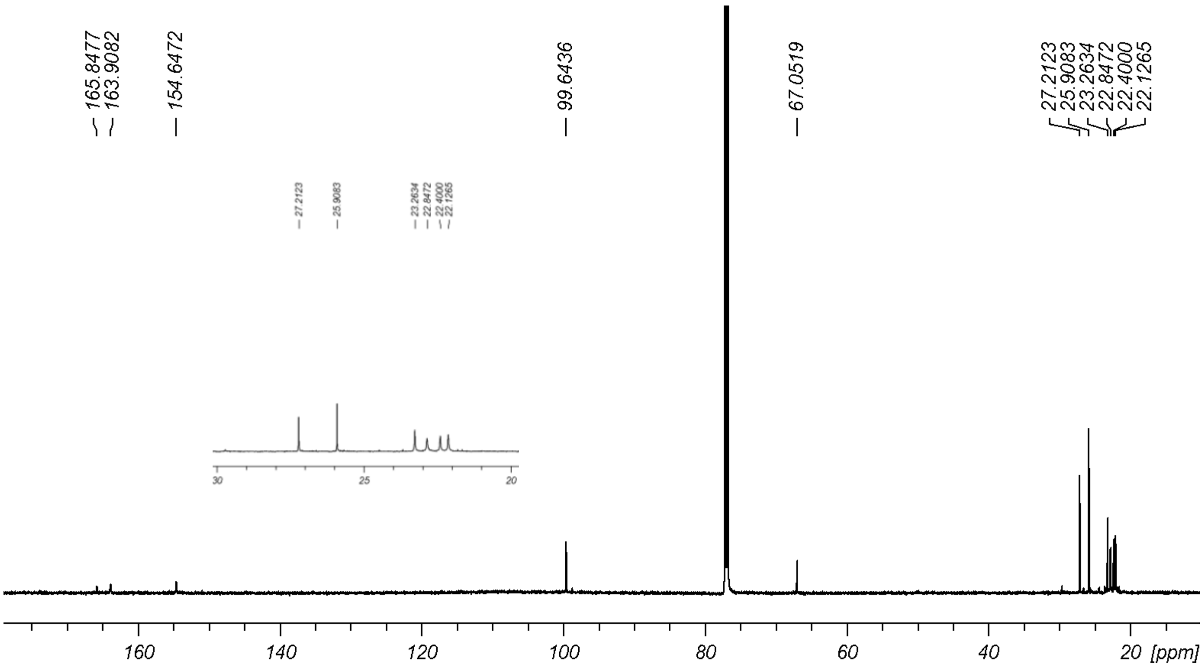

Figure S2. ^{13}C NMR spectrum of **2** in $CDCl_3$.

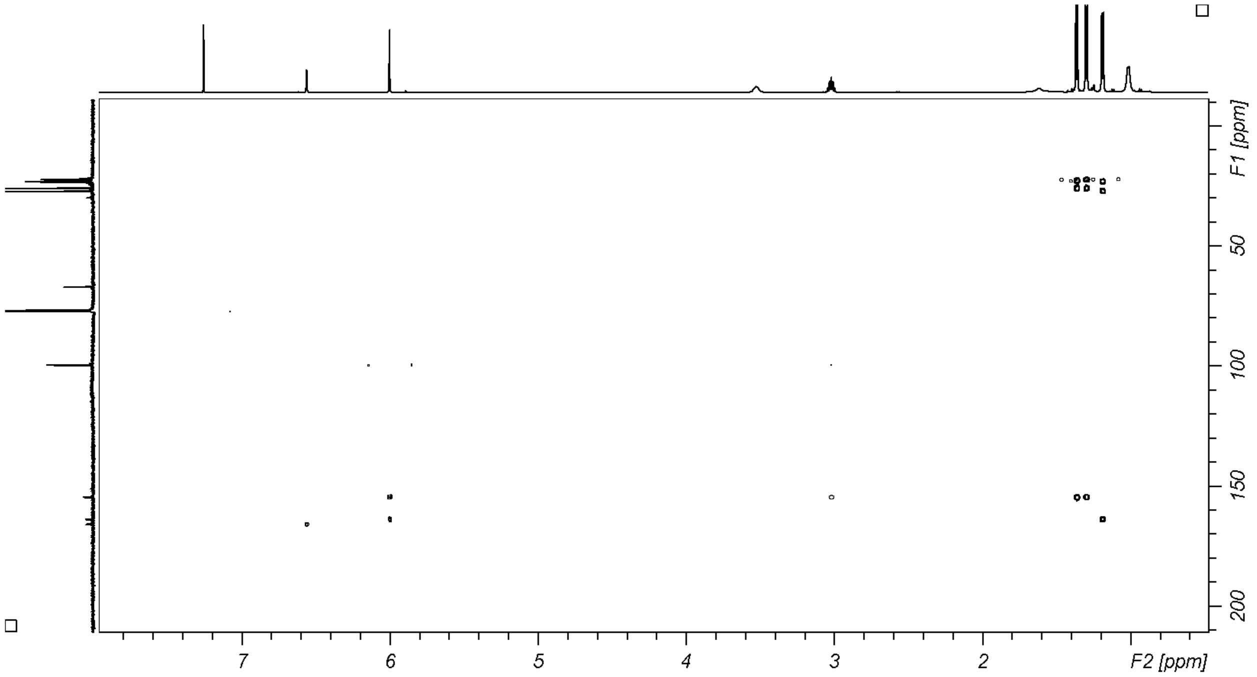

Figure S3. HMBC spectrum of **2** in $CDCl_3$.

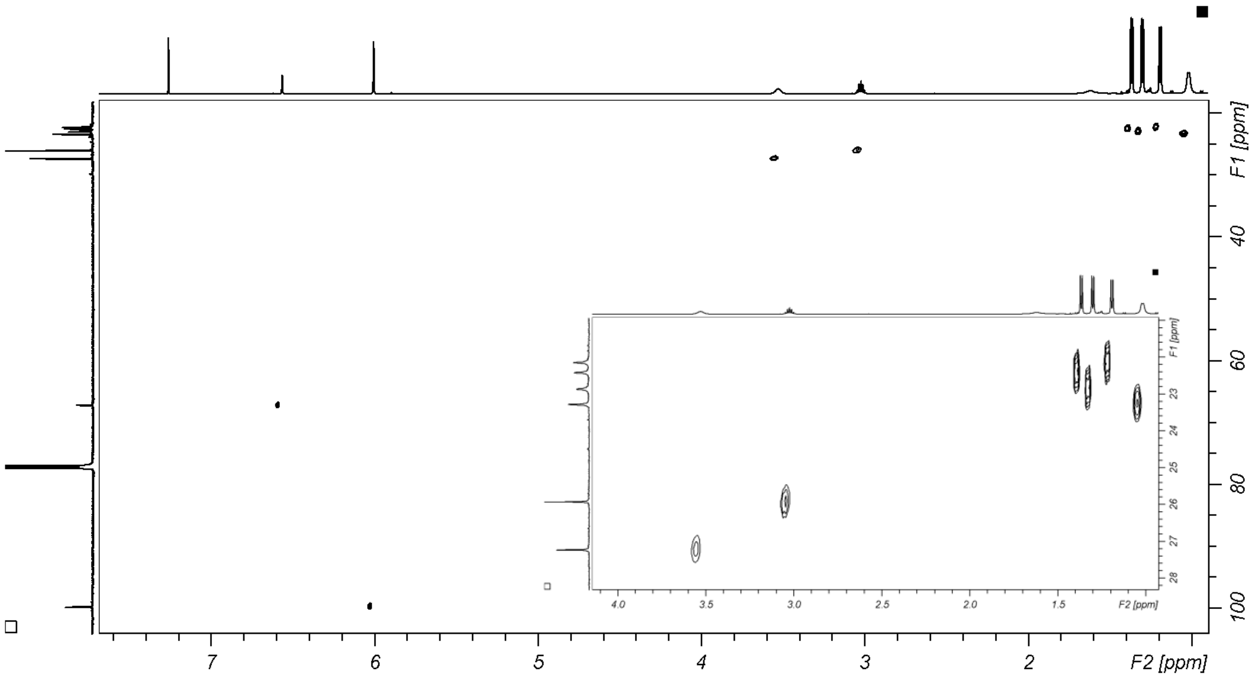

Figure S4. HSQC spectrum of **2** in $CDCl_3$. Expansion shown in bottom right.

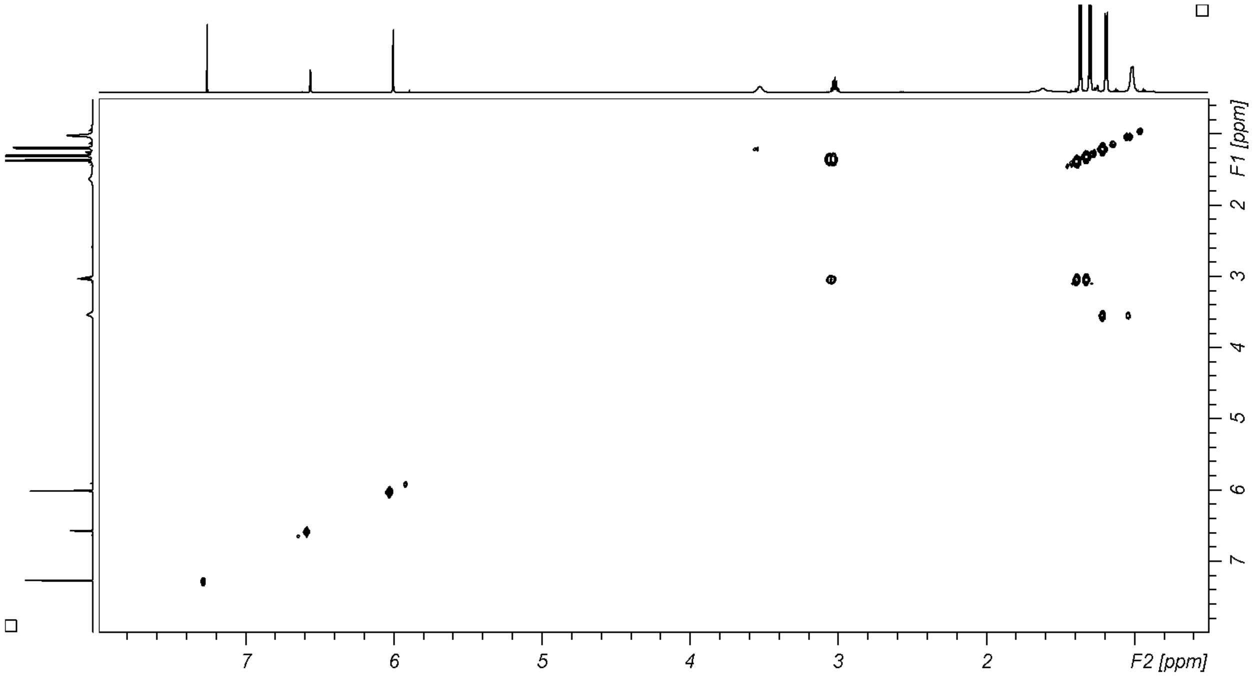

Figure S5. COSY NMR spectrum of **2** in $CDCl_3$.

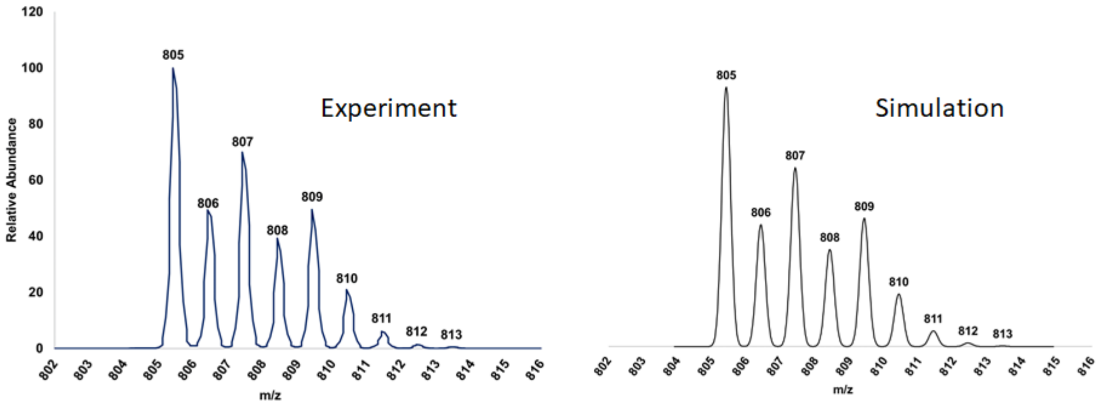

Figure S6. Experimental (left) and theoretical (right) ESI-MS spectra of **2**.
